# Ten-Year Experience with a Transapical Approach for Transcatheter Aortic and Mitral Valve Implantation

**DOI:** 10.3390/jcdd11070201

**Published:** 2024-06-29

**Authors:** Antonella Galeone, Fabiola Perrone, Gabriele Pesarini, Flavio Luciano Ribichini, Renato Di Gaetano, Giovanni Battista Luciani, Francesco Onorati

**Affiliations:** 1 Department of Surgery, Dentistry, Pediatrics and Gynecology, Division of Cardiac Surgery, University of Verona, 37126 Verona, Italy; 2 Division of Cardiology, Azienda Ospedaliera Universitaria Integrata, 37126 Verona, Italy; 3 Department of Cardiology, Azienda Sanitaria dell’Alto Adige, 39100 Bolzano, Italy

**Keywords:** transcatheter aortic valve implantation, transapical access, valve-in-valve

## Abstract

Background: The transfemoral approach represents the optimal access for TAVI due to its low invasiveness; however, up to 10–15% of TAVI candidates are considered unsuitable for femoral access because of significant peripheral vascular disease and need alternative access. Methods: This is a single-center retrospective observational study including all consecutive adult patients undergoing transcatheter procedures through a TA approach from March 2015 to April 2024. Results: 213 patients underwent transcatheter aortic or mitral valve implantation through a TA approach and were enrolled in this study. The mean age of the patients was 79.5 ± 5.7 years, and 54% of the patients were males. The mean Euroscore II was 7.9 ± 6.4%. One-third of the patients had previous cardiac surgery. The overall mean survival time was 5.3 ± 0.3 years. Nine (4%) patients developed infective endocarditis (IE) during the follow-up. Conclusions: The transapical approach for transcatheter procedures is a safe and effective procedure for patients unsuitable for TF access with low periprocedural mortality and a low rate of post-procedural complications when performed by experienced surgeons and cardiologists.

## 1. Introduction

Transcatheter aortic valve implantation (TAVI) emerged more than twenty years ago as a valid alternative to standard surgical aortic valve replacement (SAVR) for high-risk patients with severe aortic stenosis (AS) and multiple comorbidities [1]. The transfemoral (TF) approach undoubtedly represents the optimal access for TAVI, and it is also favored by current guidelines [2]. Due to its low invasiveness, TF-TAVI can be performed without general anesthesia, facilitates the patient’s recovery, and allows a shorter hospital stay. However, recent reports showed that up to 10–15% of TAVI candidates are considered unsuitable for femoral access because of significant peripheral vascular disease conditioning severe vessel narrowing, calcification, and tortuosity, or significant thoracic or abdominal aortic disease such as aneurysms and dissection [3,4]. Alternative accesses have been proposed in patients with severe iliofemoral artery disease, such as transapical (TA), transaxillary (TAx), transcarotid (TC), transaortic, transcaval and suprasternal approach [5]. TA-TAVI was first performed in 2005 [6], and it was considered the first alternative procedure when the TF approach was contraindicated [7]. The main advantages of the TA approach are fewer vascular complications, less use of contrast and fluoroscopy, a short distance from the sheath to the annulus, and improved valve alignment before deployment with fewer prosthetic valve leaks (PVLs). Disadvantages include invasiveness, risk of myocardial injury, and complications related to puncture sites, such as bleeding, ventricular apex pseudo-aneurysm, coronary artery damage, and arrhythmias [8].

Transcatheter valve technology has also been utilized in patients affected by mitral valve (MV) bioprosthesis degeneration or failed mitral valve repair (MVr) that require redo surgical mitral valve replacement (SMVR), a high-risk operation associated with high mortality and major complications [9]. Transcatheter mitral valve-in-valve (ViV-TMVI) or valve-in-ring (ViR-TMVI) implantation represents a safe and reproducible alternative for patients with a degenerated bioprosthesis or a previous MVr who are at high risk for SMVR [10]. Early experiences with ViV-TMVR were conducted via a trans-apical approach through a left mini-thoracotomy as it offers direct access and device coaxiality [11]. 

The aim of the present study was to investigate the early and long-term outcomes of patients undergoing TAVI and TMVI at our institution using a TA approach over a 10-year period since the beginning of the TA-TAVI/TVMI program in 2015.

## 2. Materials and Methods

This is a single-center retrospective observational study including all consecutive adult patients undergoing transcatheter procedures through a TA approach from March 2015 to April 2024. The study was conducted in accordance with the Declaration of Helsinki and was approved by the Ethics Committee (EC) of the Azienda Ospedaliera Universitaria Integrata of Verona. Due to the retrospective nature of the study, written informed consent was waived by the EC. Patients with symptomatic aortic and/or mitral valve disease and at high risk for surgery were screened for transcatheter valve implantation by our institutional Heart Team according to the current guidelines [2]. Patients presenting with peripheral arteries unsuitable for standard transfemoral approach at CT-scan analysis were treated using the TA approach. We enrolled in the study patients undergoing TA-TAVI for severe AS, TA-ViV-TAVI for aortic bioprosthesis degeneration, TA-ViV-TMVI for mitral bioprosthesis degeneration, and TA-ViR-TMVI for failed MVr.

Patients’ baseline characteristics, intra and perioperative data, and in-hospital outcomes were extracted from patients’ paper-based and electronic medical records. Clinical outcomes of interest included periprocedural death and postoperative complications according to the latest Valve Academic Research Consortium 3 (VARC-3) criteria [12]. Periprocedural mortality was defined as death occurring 30 days after the index procedure or >30 days but during the index hospitalization [12]. Postoperative complications included stroke, periprocedural myocardial injury (PMI), acute kidney injury (AKI), bleeding, atrioventricular block requiring permanent pacemaker (PM) implantation, valve-related complications, including conversion to surgery, implantation of multiple (>1) valves during the index hospitalization because of valve malposition, thrombosis, and paravalvular regurgitation, vascular and access site complications [12].

### 2.1. Operative Technique

All TA transcatheter procedures were performed in the catheterization laboratory by all Heart Team members, including cardiologists and cardiac surgeons. All patients underwent TA transcatheter procedures under general anesthesia, fluoroscopic control, and periprocedural transesophageal echocardiographic control. Transcatheter valve implantation was performed through a left anterolateral mini-thoracotomy at the fifth intercostal space. The left ventricular apex was prepared with two reinforced concentric 3-0 polypropylene purse-string sutures. A temporary venous PM was placed in the right ventricle for the rapid pacing through a femoral vein. During the study period, balloon-expandable Sapien XT, Sapien 3, and Sapien 3 Ultra (Edwards Lifesciences, Irvine, CA, USA) were implanted under rapid ventricular pacing. Valve sizing was determined based on preoperative ECG gathered computed tomography scan findings. After the procedure, all patients were transferred to the intensive care unit for surveillance. 

### 2.2. Follow-Up 

Follow-up data were collected until May 2024 via phone and e-mail contact with patients, family members, family physicians, and cardiologists. Subsequent hospitalization and routine visit data were collected from hospital records and cardiology reports. The follow-up time was calculated either to death or to the last verified contact with the patient. Clinical outcomes of interest included mortality and bioprosthetic valve dysfunction (BVD). Mortality was defined according to Valve Academic Research Consortium 3 (VARC-3) as early (occurring >30 days but ≤1 year after the index hospitalization) and late mortality (occurring >1 year after the index hospitalization) according to VARC-3 criteria [12]. BVD was defined as the presence of structural valve dysfunction (SVD), non-SVD (NSVD), infective endocarditis, and thrombosis [12]. 

### 2.3. Statistical Analysis

Categorical variables are expressed as numbers and percentages and compared with the χ^2^ test. Continuous variables with a skewed distribution are presented as median and interquartile range and compared with the Mann–Whitney U test. The Kaplan–Meier method was used to draw survival curves; the log-rank test was used to compare survival among groups. The Reverse Kaplan–Meier method was used to calculate the median follow-up time. 

## 3. Results

During the study period, 213 consecutive patients underwent transcatheter aortic or mitral valve implantation through a TA approach at our institution and were enrolled in the study. The median age of the patients was 80 (77–84) years (mean: 79.5 ± 5.7 years), and 54% of the patients were males. Median Euroscore II was 5.68 (3.55–1.46)% (mean: 7.9 ± 6.4%). One-third of the patients had previous cardiac surgery. The baseline characteristics of the patients are illustrated in Table 1.

One hundred sixty-four (77%) patients underwent TA-TAVI for severe aortic stenosis, 10 (5%) patients underwent TA-ViV-TAVI for aortic prosthesis degeneration, 30 (14%) patients underwent TA-ViV-TMVI for mitral prosthesis degeneration, 6 (3%) patients underwent both TA-TAVI and ViV-TMVI for severe AS and mitral prosthesis degeneration and 3 (1%) patients underwent TA ViR-TMVI for MVr failure. A total of 219 prostheses were implanted in 213 patients. Details of implanted prostheses and intra and periprocedural outcomes are illustrated in Table 2.

### 3.1. Survival

**Figure 1:**
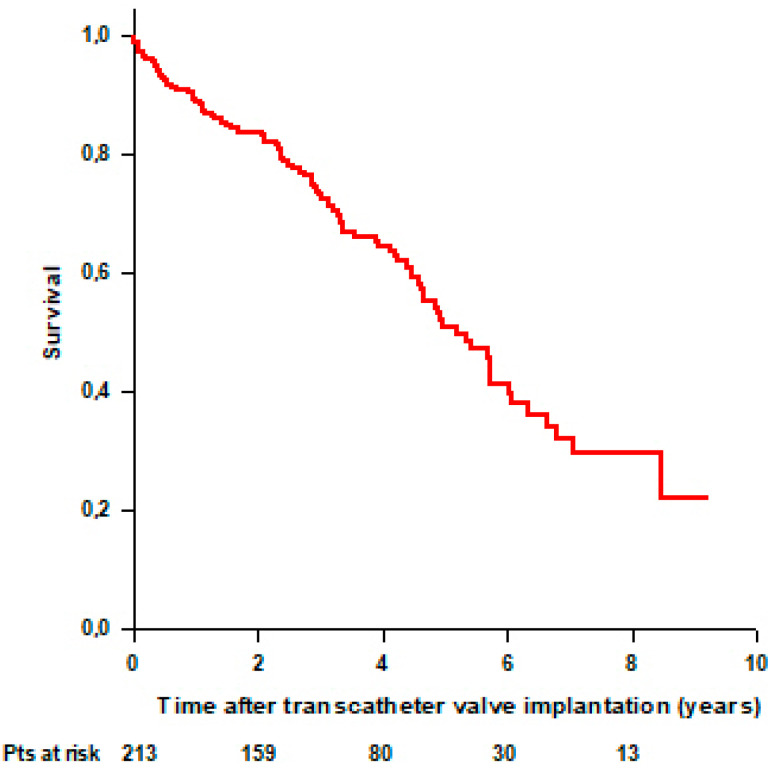
Overall patients’ survival after transcatheter aortic and mitral valve implantation through TA approach.

Follow-up was 100% complete, and the mean follow-up time was 4.7 ± 0.2 years. There was no intra-procedural death. Ninety-one (42.7%) patients died during the follow-up. We recorded 6 (2.8%) periprocedural deaths, 17 (7.9%) early deaths, and 68 (31.9%) late deaths. Periprocedural deaths occurred in 4 patients undergoing TA-TAVI for AS and 2 patients undergoing TA-ViV-TAVI for aortic prosthesis degeneration. Causes of periprocedural death were valve thrombosis (*n* = 1), stroke (*n* = 2), sepsis (*n* = 1), cardiac arrest (*n* = 1), and bowel ischemia (*n* = 1). The overall mean survival time was 5.3 ± 0.3 years. Survival rates were 98.6% at 30 days, 89% at 1 year, and 51% at 5 years (Figure 1).

**Figure 2:**
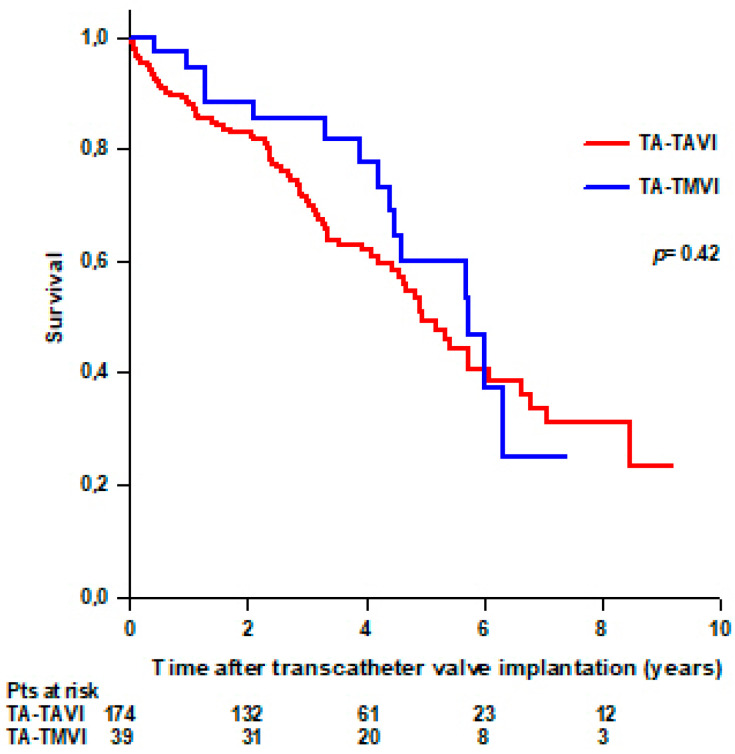
Survival after transcatheter valve implantation through TA approach in patients undergoing TA-TAVI and patients undergoing TA-TVMI.

No difference was observed in survival between patients who underwent TA-TAVI and patients who underwent TA-TVMI. Seventy-six (44%) patients with TA-TAVI and 15 (38%) patients with TA-TMVI died during the follow-up. Mean survival was 5.2 ± 0.3 years in patients with TA-TAVI and 5.2 ± 0.4 years in patients with TA-TMVI (*p* = 0.42) (Figure 2).

**Figure 3:**
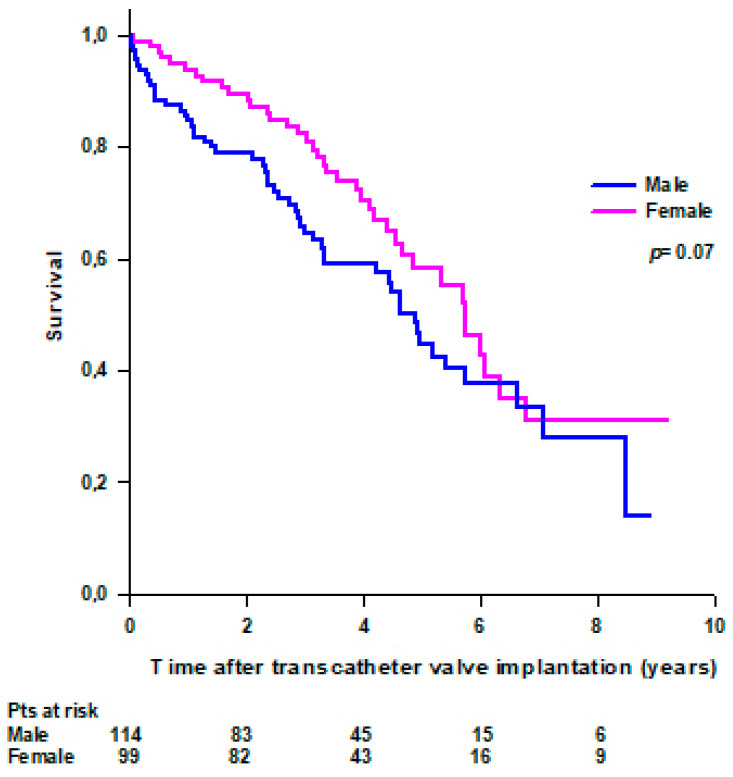
Survival after transcatheter valve implantation through TA approach in male and female patients.

Fifty-three (46%) male and 38 (38%) female patients died during the follow-up. No difference was observed in survival between male and female patients, and mean survival was 4.8 ± 0.4 years for male and 5.7 ± 0.4 years for female patients (*p* = 0.07) (Figure 3).

**Figure 4:**
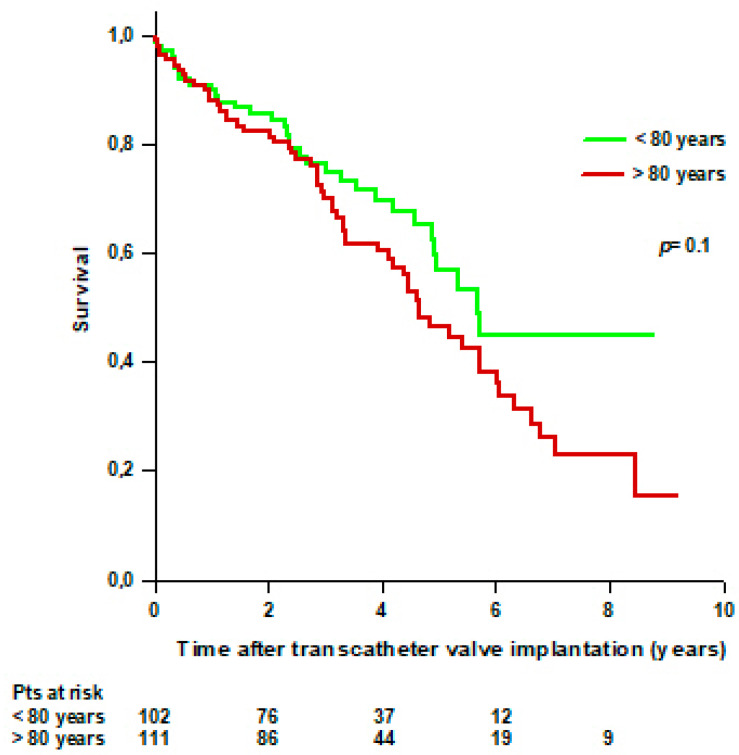
Survival after transcatheter valve implantation through TA approach in patients aged <80 years and >80 years.

There was no difference in survival between patients aged < 80 years and patients aged > 80 years at the time of transcatheter valve implantation. Thirty-three (32%) patients aged < 80 years and 58 (52%) patients aged > 80 years died during the follow-up. The mean survival time was 5.8 ± 0.4 years for patients aged < 80 years and 4.9 ± 0.3 years for patients aged > 80 years (*p* = 0.1) (Figure 4).

**Figure 5:**
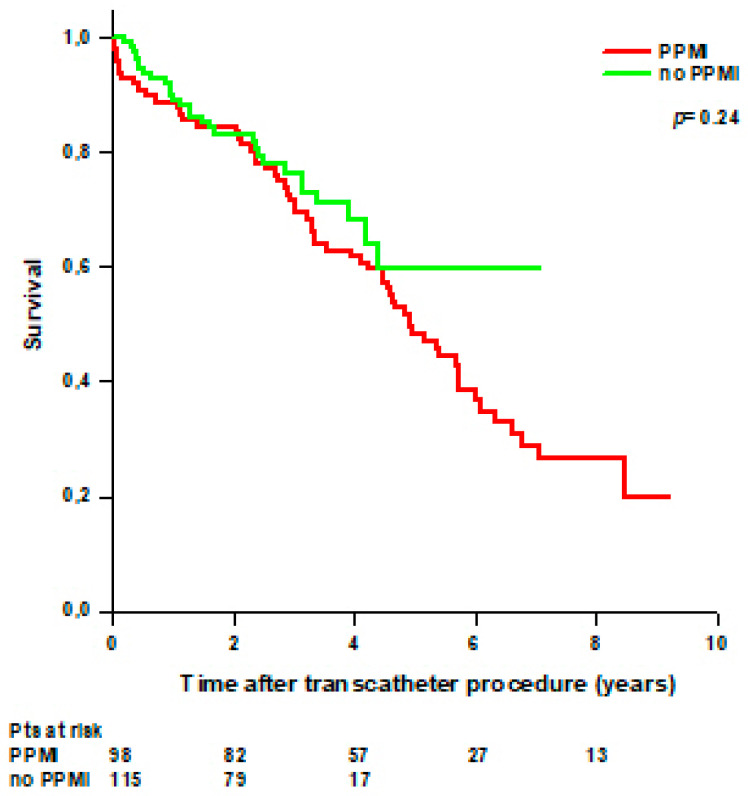
Survival after transcatheter valve implantation through TA approach in patients with and without periprocedural myocardial injury (PPMI).

More patients with PPMI died during the follow-up (62/98, 63%) compared with patients without PPMI (29/115, 25%); however, no statistically significant difference was observed in survival between patients with PPMI and patients without PPMI (Figure 5).

### 3.2. Follow-Up Events

**Figure 6:**
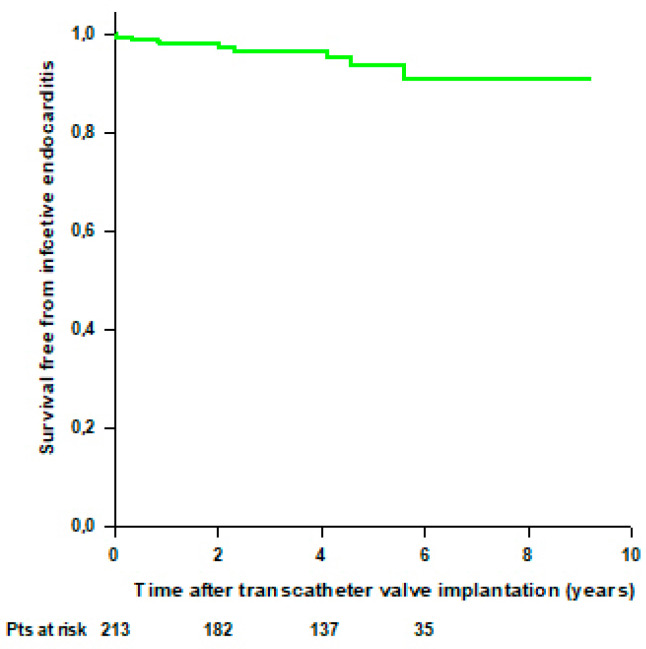
Survival free from infective endocarditis after transcatheter valve implantation through TA approach.

Nine (4%) patients developed infective endocarditis (IE) during the follow-up; 4 patients had early IE (within 1 year from the transcatheter procedure), and 5 patients developed late IE (>1 year following the transcatheter procedure). Infective endocarditis was recorded in 7 patients with TA-TAVI, 1 patient with TA-ViV-TAVI, and 1 patient with TA-ViV-TMVI. Two patients underwent open heart surgery for IE and were still alive at the end of the follow-up, while 7 patients were treated with ev antibiotics and died during the follow-up. Survival rates free from IE were 97.9% at 1 year and 93.7% at 5 years (Figure 6). One patient with previous TA-ViV-TMVR underwent TF-TAVI for severe AS. There was no re-operation for SVD during the follow-up period.

## 4. Discussion

Our series showed that transcatheter valve implantation through a TA approach is a safe procedure for patients unsuitable for TF access with periprocedural mortality of 2.8% and a low rate of post-procedural complications when performed by an experienced multidisciplinary heart team. Previously published series reported higher periprocedural death rates in patients undergoing TA-TAVI ranging from 4% to 15.7%, with contradictory findings when compared with a TF approach [13]. In the PREVAIL transapical study, including 150 patients undergoing TA-TAVI using the Sapien XT valve the mortality at 30 days was 8.7% [13]. A French registry enrolling 3195 patients showed that 30-day mortality was significantly lower with the TF approach than with the TA approach (8.5% vs. 13.9%, respectively) [14]. Similarly, a study on 1620 patients undergoing TAVI in the UK showed that TA access was associated with significantly higher 30-day mortality compared with TF access (11.2% vs. 4.4%, respectively) [15]. Another observational study of 4 European registries also found lower 30-day mortality in patients undergoing TF-TAVI compared to patients undergoing TA-TAVI (6.4% vs. 15.7%, respectively) [16]. A substudy of the Placement of Aortic Transcatheter Valves (PARTNER)-I trial compared outcomes of 501 propensity-score (PS) matched pairs of TF and TA-TAVI procedures and found that TF-TAVI was associated with significantly lower rates of in-hospital mortality compared with TA-TAVI (2.8% vs. 7.4% respectively) [17]. Another PS-matched analysis of 1576 pairs of TF-TAVI and TA-TAVI procedures showed that TF-TAVI was associated with significantly lower in-hospital mortality (3.1% vs. 4.9%, respectively) [18].

In contrast to these findings, a PS-matching analysis of two groups of 354 patients, each with either access route, found no significant difference in 30-day mortality for TA and TF access (5.9% versus 8.5% respectively [19]. Similarly, a PS-matched analysis of 199 pairs of TF and TA-TAVI procedures from an Italian registry showed no association between access choice and short-term mortality 4% for TF-TAVI and 8% for TA-TAVI) [20]. A single-center study from a large volume center in the United States also showed that all-cause mortality at 30 days was identical in both TF and TA groups (4.5% vs. 5.3%, respectively) [21]. Another study found similar hospital mortality between the TF and TA approach (10% vs. 9% respectively) despite the fact that the logistic Euroscore was significantly higher in the TA group [22].

The TC approach has emerged since 2010 as a safe alternative in patients with unfavorable ilio-femoral artery anatomy. However, stroke remains the main concern for the TC approach. A recent metanalysis including 22 observational studies with a total of 11,896 patients showed that the TC approach reduced mortality and the risk of major vascular complications and major bleeding compared with the TA approach, but the difference was not statistically significant. Additionally, the TC approach did not increase the risk of stroke compared with TF or the other alternative accesses [23]

A recent study focused on 9686 patients who received a non-TF access and compared TA approach to other alternative vascular accesses and found that TA patients had a significantly lower 30-day survival compared to TAx approach patients (TA 90.92% vs. TAx 95.59%) [24]. A recent meta-analysis analyzed intrathoracic (IT: TA and transaortic) and extrathoracic (ET: TC, TAx, and transubclavian) vascular accesses for TAVI in patients in which the TF approach was contraindicated. The metanalysis included 18 studies with 6800 IT-TAVR patients and 5032 ET-TAVR patients. IT access was associated with a significantly higher risk of in-hospital or 30-day all-cause mortality (RR 1.99, 95% CI: 1.67 to 2.369) [25]. 

Previous studies suggested that in an experienced multidisciplinary heart team, either access route can be performed with comparable results [19]. A recent report on 1130 patients scheduled for TAVI showed that TA patients had a higher operative risk profile compared to TF patients (logistic EuroSCORE: 24% vs. 17%). The unadjusted 30-day mortality rate was higher in TA than in TF patients, albeit this difference was not significant [TA: 6.7%, TF: 4.8%]. The multivariate logistic regression analysis revealed the logistic EuroSCORE and institutional experience but not the access mode as independent predictors of 30-day mortality [26]. In institutions performing a low volume of TA-TAVI, the technique is associated with an increased risk of all-cause mortality and longer hospital stays but fewer vascular complications in comparison with TF-TAVI. Besides patient-related variables, it has been suggested that procedural and operator-related factors may play an important role in the observed difference in outcome. TA-TAVI is usually performed in fewer patients thus conditioning a dissimilar experience and expertise between TF-TAVI and TA-TAVI. Local expertise seems to be the most important factor of favorable results in TA TAVI. Centers performing higher volumes of TA procedures demonstrate more favorable results, suggesting a clear relationship between volume and outcomes. In a series reporting the results of ten-year experience in 312 consecutive high-risk patients treated with TA-TAVI, 30-day mortality decreased from 8.2% to 4.2% in later years, suggesting that time and practice contribute to successful outcomes [27]. Additionally, a trend towards lower mortality after TA-TAVI similar to that observed after TF-TAVI in more recent studies could reflect the improved experience of investigators as well as advances in device technology and perioperative management.

Postprocedural myocardial injury or infarction has been observed in up to two-thirds of patients after transcatheter procedures [28,29] and is associated with worse outcomes [30,31]. In our series, we found that 46% of patients had PPMI after a transcatheter procedure using a troponin threshold to define PPMI as an increase > 70 times the local laboratory upper reference limit (URL) according to the latest VARC-3 criteria [12]; however, we did not find any difference in short and long-term survival between patients with and without PPMI. Conversely, a recent report on 1394 consecutive patients who underwent TAVI found that only 14.0% of patients had PPMI according to VARC-3 criteria and that PPMI was associated with a higher risk of all-cause mortality at 30-day and 1-year [32]. 

Renal complications occurred in only 23% of patients in our series, and we observed mostly AKI stage 1 and no case of AKI stage 4. In contrast, the previous report reported a higher incidence of AKI in patients undergoing TA-TAVI, up to 66.7% [33]. Despite being performed with less contrast, TA-TAVI is associated with a higher risk of AKI, and it has been proposed that surgical trauma, systemic inflammatory response, and renal damage could contribute to the development of AKI [33]. Additionally, some reports showed that TF-TAVI was associated with lower rates of AKI when compared with TA-TAVI [17], while others did not [21]. 

Another emerging and serious complication after transcatheter procedures is IE. Previous studies showed that IE after TAVI has an incidence of 0.3 to 2.0 per 100 person-years, which is similar to that observed after SAVR [34,35,36,37]. It has also been suggested that early IE after TAVI is more common than late IE after TAVI [38]. However, a meta-analysis of the most relevant randomized controlled trials comparing TAVR and SAVR found no differences in early and late IE incidence between the groups [39]. Consistently, in our series, 4% of patients developed IE during the follow-up, and we found a similar rate of early and late IE. Surgical treatment is associated with a large survival advantage in patients with prosthetic valve endocarditis, even if it implies substantial operative mortality [40,41,42]. However, previous studies reported low surgical rates in patients with IE after transcatheter procedures around 20% [43,44,45] that are similar to that reported in this study. Few studies have compared surgery and medical therapy alone in patients with IE after TAVI, showing that cardiac surgery was not associated with improved in-hospital mortality or all-cause mortality at 1 year compared with medical management alone [46,47,48]. Specific guidelines for the management of patients with IE after transcatheter procedures are strongly required and further studies are needed to determine the benefit of surgery in these patients.

The main limitation of this study is that it is a single-center, retrospective observational study on a small population, and selection bias is unavoidable. Additionally, pre and postoperative management of the patients could have changed during the last decade. Therefore, the results of our series cannot be generalized to the entire population, and multicenter studies are needed to provide more precise information.

## 5. Conclusions

Transcatheter aortic and mitral valve implantation through a TA approach is a safe and effective procedure for patients unsuitable for TF access with low periprocedural mortality and a low rate of post-procedural complications when performed by experienced surgeons and cardiologists.

## Figures and Tables

**Table 1:** Patients’ baseline characteristics.

Characteristics	Overall (*n* = 213)
Age (years)	80 (77–84)
Sex, male	114 (54%)
Body mass index	26.4 (23.1–29.8)
Body surface area (m^2^)	1.8 (1.7–1.9)
Hypertension	178 (84%)
Diabetes	66 (31%)
Dyslipidemia	124 (58%)
Previous Acute myocardial infarction	34 (16%)
Previous Percutaneous coronary intervention	36 (17%)
Heart failure	37 (17%)
Pacemaker	30 (14%)
Previous cerebrovascular accident	34 (16%)
Chronic obstructive pulmonary disease	76 (36%)
Hemodialysis	3 (1%)
Peripheral artery disease	123 (58%)
Hepatic cirrhosis	14 (7%)
Poor mobility	50 (23%)
Hostile chest	38 (18%)
Porcelain aorta	80 (38%)
Mediastinal irradiation	11 (5%)
Previous cardiac surgery	70 (33%)
Aortic valve replacement	15 (7%)
Mitral valve replacement	40 (19%)
Coronary artery bypass grafting	28 (13%)
Mitral valve repair	3 (1%)
Ascending aorta replacement	2 (1%)
Aortic valve repair	1 (0.5%)
Euroscore II (%)	5.68 (3.55–11.46)
Left ventricular ejection fraction (%)	60 (50–64)
Peak aortic valve gradient (mmHg)	67 (53–76)
Mean aortic valve gradient (mmHg)	41 (30–47)
Aortic valve area (cm^2^)	0.8 (0.6–0.9)
Maximum aortic annulus diameter (mm)	27 (25–28)
Minimum aortic annulus diameter (mm)	21 (19–23)
Aortic annulus area (mm^2^)	447 (402–512)
Aortic annulus perimeter (mm^2^)	76 (72–80)
Sino-tubular junction diameter (mm)	28 (26–31)
Systolic Pulmonary artery pressure (mmHg)	43 (35–58)
Moderate/severe Pulmonary artery Hypertension	22 (10%)

**Table 2:** Intra and periprocedural characteristics.

Characteristics	Overall (*n* = 213)	TA-TAVI (*n* = 180)	TA-TMVI (*n* = 39)
TA-TAVI	164 (77%)	164 (91%)	-
TA-ViV-TAVI	10 (6%)	10 (6%)	-
TA ViV-TMVI	30 (14%)	-	30 (77%)
TA-TAVI + ViV-TMVI	6 (3%)	6 (3%)	6 (15%)
TA-ViR-TMVI	3 (1%)	-	3 (8%)
Valve type			
Sapien 3	124 (57%)	102 (57%)	22 (56%)
Sapien 3 Ultra	88 (40%)	75 (42%)	13 (33%)
Sapien XT	7 (3%)	3 (2%)	4 (10%)
Valve size	26 (23–29)	26 (23–26)	29 (26–29)
Valve size distribution			
20 mm	2 (1%)	2 (1%)	0 (0%)
23 mm	60 (27%)	59 (33%)	1 (3%)
26 mm	96 (44%)	86 (48%)	10 (26%)
29 mm	61 (29%)	33 (18%)	28 (72%)
Procedural time (min)	106 (88–128)	107 (88–128)	105 (94–129)
Valve malposition	0 (0%)	0 (0%)	0 (0%)
Valve thrombosis	1 (1%)	1 (1%)	0 (0%)
LVOT obstruction	0 (0%)	0 (0%)	0 (0%)
Intraprocedural PCI	8 (4%)	8 (4%)	0 (0%)
Non-planned intraprocedural PCI	4 (2%)	4 (2%)	0 (0%)
Staged PCI during the index hospitalization	11 (5%)	10 (6%)	1 (3%)
Coronary artery obstruction	5 (2%)	5 (3%)	0 (0%)
Aortic dissection	0 (0%)	0 (0%)	0 (0%)
Cardiac tamponade	2 (1%)	2 (1%)	0 (0%)
Conversion to sternotomy	1 (1%)	1 (1%)	0 (0%)
Pacemaker implantation	6 (3%)	5 (3%)	1 (3%)
Re-exploration for bleeding	6 (3%)	3 (2%)	3 (8%)
Cerebro-vascular accident	2 (1%)	1 (1%)	1 (3%)
Vascular complication	11 (5%)	9 (5%)	2 (5%)
Mechanical ventilation, hours	8 (6–8)	8 (6–8)	6 (5–8)
Prolonged mechanical ventilation > 24 h	5 (2%)	5 (3%)	0 (0%)
Intensive Care Unit stay, days	1 [1–2]	1 [1–2]	1 [1–3]
Hospital stay, days	7 [6–9]	7 [5–9]	7 [6–10]
Troponin peak (ng/L)	937 [483–5932]	989 [505–6246]	686 [418–5032]
Periprocedural myocardial injury	98 (46%)	84 (47%)	14 (36%)
Acute kidney injury	48 (23%)	39 (22%)	9 (23%)
Stage 1	41 (19%)	33 (18%)	8 (20%)
Stage 2	2 (1%)	2 (1%)	0 (0%)
Stage 3	5 (2%)	4 (2)	1 (3%)
Stage 4	0 (0%)	0 (0%)	0 (0%)
Bowel ischemia	1 (1%)	1 1%)	0 (0%)
Red blood cell transfusion	54 (25%)	41 (23%)	13 (33%)
Left ventricular ejection fraction at discharge (%)	56 (50–60)	60 (55–60)	53 (45–60)
Aortic Perivalvular leak at discharge	23 (11%)	23 (13%)	-
Mild	21 (10%)	21 (12%)	-
Moderate	2 (1%)	2 (1%)	-
Severe	0 (0%)	0 (0%)	-
Mitral Perivalvular leak at discharge	5 (2%)	-	5 (13%)
Mild	3 (1%)	-	3 (8%)
Moderate	2 (1%)	-	2 (5%)
Severe	0 (0%)	-	0 (0%)
Intra-procedural mortality	0 (0%)	0 (0%)	0 (0%)
In-hospital mortality	3 (1%)	3 (2%)	0 (0%)

## Data Availability

The data presented in this study are available on request from the corresponding author.
